# Chronic Subdural Hematoma as a Potentially Reversible Cause of Cognitive and Gait Decline in Older Adults: Clinical Recognition, Treatment, and Functional Recovery

**DOI:** 10.3390/jcm15218605

**Published:** 2026-11-04

**Authors:** Nikodem Kuczyński, Mariusz Sowa, Mariola Głowacka, Edyta Zomkowska, Dawid Pilewski

**Affiliations:** 1 Faculty of Medicine, Collegium Medicum, The Mazovian University in Płock, 09-402 Płock, Poland; 2 Department of Neurology and Neurosurgery, Faculty of Medicine, Collegium Medicum, University of Warmia and Mazury in Olsztyn, Aleja Warszawska 30, 10-082 Olsztyn, Poland; neurochirurgia@uwm.edu.pl; 3 Department of Nursing, Faculty of Health Sciences, Collegium Medicum, The Mazovian University in Płock, 09-402 Płock, Poland; m.glowacka@mazowiecka.edu.pl; 4 Department of Neurosurgery, Neurotraumatology and Spinal Surgery, Regional Hospital in Elblag, 82-300 Elblag, Poland; ezomkowska14@gmail.com; 5 Faculty of Health Science, Collegium Medicum, The Mazovian University in Płock, 09-402 Płock, Poland

**Keywords:** chronic subdural hematoma, cognitive impairment, gait disturbance, older adults, rehabilitation, early mobilization, therapeutic exercise, middle meningeal artery embolization, frailty, functional recovery

## Abstract

Chronic subdural hematoma (cSDH) is an increasingly common neurological disorder in older adults and may present with cognitive decline, gait instability, recurrent falls, and loss of independence rather than overt focal deficits. Because these symptoms overlap with dementia, delirium, parkinsonism, and frailty, diagnosis may be delayed despite the potential for meaningful recovery after treatment. This narrative review summarizes evidence on clinical recognition, treatment, early mobilization, rehabilitation, and patient-centered recovery in older adults with cSDH. A targeted search of PubMed/MEDLINE, Scopus, and Web of Science identified randomized trials, observational studies, systematic reviews, meta-analyses, guidelines, and consensus statements available through July 2026. Surgical decompression may improve cognition and mobility, but the magnitude and durability of recovery vary. Premorbid cognitive and functional status, frailty, malnutrition, postoperative delirium, and complications are associated with outcomes, although causal effects are incompletely established. Postoperative drainage reduces recurrence; selected randomized trials suggest that middle meningeal artery embolization reduces recurrence or treatment failure, whereas evidence for independent cognitive and gait benefits remains limited. Early mobilization may reduce medical complications. Evidence specific to cSDH remains insufficient to prescribe a standardized exercise dose or progression. Multidimensional follow-up and prospective trials using cognitive, gait, quality-of-life, and participation outcomes are needed to determine whether successful hematoma treatment translates into restored independence and meaningful daily function.

## 1. Introduction

Chronic subdural hematoma (cSDH) is a common neurosurgical disorder in older adults. Its incidence has increased substantially over recent decades. A Finnish population-based study showed that the incidence more than doubled between 1990 and 2015. The largest increase was observed in people aged 80 years or older [1]. Contemporary data from the United States suggest an annual incidence of approximately 17 cases per 100,000 people [2]. As populations continue to age, the number of patients with cSDH is also likely to rise. Antiplatelet and anticoagulant drug use is also common among surgically treated patients and introduces additional perioperative management considerations [3]. Earlier North American projections and epidemiological reviews similarly anticipated a steep age-related increase in cSDH and emphasized its growing health-system burden [4,5]. A large Danish case–control study further showed that antithrombotic exposure, particularly combined regimens, is associated with an increased risk of subdural hematoma [6]. An influential earlier review described cSDH as a disorder influenced by ageing, cerebral atrophy, trauma, and antithrombotic exposure [7].

The symptoms of cSDH often develop slowly and may vary between patients. Common features include headache, focal neurological deficits, confusion, cognitive decline, gait instability, and recurrent falls [8]. In a large Swiss multicenter study, gait disturbance was reported in more than half of the patients. Cognitive deterioration was present in approximately 40% [9]. These symptoms are not always recognized as signs of an intracranial disorder. In older adults, they may initially be explained by normal aging, frailty, dementia, depression, or medication use. For this reason, cSDH should be considered in patients with new or progressive cognitive and gait problems [2,9].

The clinical importance of cSDH is partly related to the possibility of cognitive and neurological recovery after treatment [10]. The hematoma can compress the brain and disturb the function of nearby structures. This may affect attention, memory, mobility, and postural control. Earlier studies showed that surgical evacuation can improve dementia-like symptoms and independence in daily activities [11]. Postural stability may also improve after surgery [12]. These findings suggest that cognitive and gait decline caused by cSDH may be at least partly reversible. Early diagnosis is therefore important, especially when symptoms have developed over a short period [11,12].

However, improvement is not always complete. Some patients continue to experience cognitive or functional problems after successful treatment. In one study, more than half of the patients had cognitive scores consistent with impairment three months after treatment [13]. Long-term survivors have also shown lower functional independence and poorer performance in selected memory domains. Reduced psychological and social well-being has also been reported [14]. Recent evidence supports these concerns. A 2026 population-matched study found persistent cognitive and functional limitations approximately ten years after surgery. The patients also had lower long-term survival than the matched general population [15]. cSDH should therefore not be viewed only as a temporary surgical problem.

Most studies of cSDH have focused on mortality, recurrence, surgical complications, and radiological outcomes. Global disability scales are also commonly used. These outcomes are important, but they do not describe every aspect of recovery. Global disability scores may fail to capture cognitive deficits, unsafe gait, and restricted participation even when the clinical outcome is conventionally classified as favorable. Cognitive function, gait, daily activities, and quality of life should therefore receive greater attention. This narrative review summarizes current evidence on cSDH as a potentially reversible cause of cognitive and gait decline in older adults. Its distinct contribution is a clinically usable assessment framework integrating cognitive, gait, functional, and participation outcomes while distinguishing trial-supported interventions from proposals extrapolated from general geriatric rehabilitation.

Although multidisciplinary and Improving Care in Elderly Neurosurgery Initiative (ICENI) guidelines [16,17] provide recommendations for the management of chronic subdural hematoma, and recent systematic reviews have examined cognitive impairment [18] and broader health-related outcomes [19], the integration of cognitive, gait, mobility, and functional recovery into clinical assessment remains insufficiently explored. This narrative review addresses this gap by examining the distinction between hematoma-related deficits and pre-existing cognitive or functional impairment, the variability of recovery across different domains, and the clinical applicability of assessment tools. Particular emphasis is placed on evaluating recovery beyond radiological resolution and global disability scores, incorporating cognition, balance, mobility, independence in daily activities, and social participation. By integrating these dimensions, the review aims to provide a practical, patient-centered framework for assessing recovery and guiding longitudinal follow-up in older adults with cSDH.

## 2. Literature Search Strategy

This article was prepared as a narrative review of the literature on cSDH in older adults, with particular emphasis on cognitive and gait impairment, clinical recognition, treatment, early mobilization, rehabilitation, and functional recovery. A targeted, non-systematic search of PubMed/MEDLINE, Scopus, and Web of Science identified relevant publications available through July 2026. The core Boolean concept was (“chronic subdural hematoma” OR “chronic subdural haematoma” OR cSDH) AND (“cognitive impairment” OR cognition OR gait OR balance OR mobility OR “functional recovery” OR rehabilitation OR “middle meningeal artery embolization”).

Randomized controlled trials, observational studies, systematic reviews, meta-analyses, clinical guidelines, consensus statements, and relevant narrative reviews were considered. Selected case reports were included when they illustrated clinically important or atypical presentations. Human studies published in English were prioritized; one French-language review was also included because of its direct relevance to cSDH in older adults. Animal studies, pediatric studies, publications focused exclusively on acute subdural hematoma, and conference abstracts without available full texts were excluded.

Studies were selected purposively for direct relevance to older adults, measurable cognition or mobility, comparative treatment evidence, or pragmatic assessment and follow-up. When evidence was overlapping or inconsistent, priority was given to recent randomized trials, multicenter studies, larger cohorts, systematic reviews, and clinical guidelines. Reference lists of relevant publications were additionally examined to identify further studies.

The findings were synthesized qualitatively according to the main clinical topics covered in the review. Because of the narrative design, no formal risk-of-bias assessment, meta-analysis, or quantitative synthesis was performed.

## 3. Cognitive and Gait Decline in cSDH

Cognitive impairment and gait disturbance are common manifestations of cSDH and may occur together, particularly in older adults [20]. Both can reduce independence and contribute to an increased risk of falls. Although cognitive and mobility-related symptoms may improve after treatment, the extent and timing of recovery vary considerably between patients [21]. Cognition, balance, and mobility should therefore be assessed as distinct clinical outcomes rather than inferred solely from radiological improvement or a global disability score [22].

### 3.1. Cognitive Impairment

Cognitive impairment in cSDH is not a single, uniform syndrome. Neuropsychiatric and neuropsychological manifestations may include psychomotor slowing, impaired attention and memory, disorientation, apathy, and behavioral changes [23]. In some patients, cognitive or psychiatric symptoms may predominate without an obvious focal motor deficit [24]. Consequently, cSDH may resemble dementia, delirium, depression, or another neurodegenerative or psychiatric disorder [23,24].

In a retrospective cohort of 356 surgically treated patients, Schebesch et al. [25] reported memory deficits, cognitive decline, or confusion in 192 of 356 patients (53.9% of the complete cohort). The authors also warned that neuroimaging may be delayed when these symptoms are initially attributed to dementia [25]. The combination of recent cognitive deterioration, falls, and new gait impairment should prompt consideration of a structural cause [16].

The most comprehensive estimate of cognitive impairment in cSDH comes from the systematic review by Blaauw et al. [18]. The review included 22 studies and separated subjective cognitive complaints from impairment confirmed by cognitive testing. The pooled prevalence of subjective complaints or objectively assessed cognitive impairment at presentation was 45% (95% confidence interval [CI], 36–54%). In four prospective studies, objectively measured cognitive impairment decreased from 61% (95% CI, 51–70%) before treatment to 18% (95% CI, 8–32%) after surgery. These results support the potential for cognitive recovery. However, they should be interpreted carefully. The included studies used different definitions and cognitive tests. Post-treatment assessment was often performed within 24 h to two weeks, and premorbid cognitive status was usually unknown [18].

Cognitive symptoms may also carry prognostic information. In an observational multicenter cohort of 1307 patients, cognitive complaints were present in almost one-third of cases. They were independently associated with a worse functional outcome at follow-up. This association remained after adjustment for age, comorbidity, hematoma characteristics, treatment, recurrence, and postoperative complications [26]. Cognitive status in this study was based mainly on symptoms recorded in medical files rather than formal testing. Despite this limitation, the findings suggest that cognitive complaints identify a clinically vulnerable group. They should not be treated as a minor or nonspecific feature of cSDH.

A practical cognitive assessment should establish premorbid functioning through a relative or caregiver, then distinguish pre-existing impairment, acute hematoma-related deficits, delirium, and possible neurodegenerative disease. Brief tests can detect impairment, but the Mini-Mental State Examination (MMSE) is relatively insensitive to executive dysfunction and subtle frontal-subcortical deficits; executive tasks such as verbal fluency or Trail Making Test performance may add clinically useful information when feasible. Reassessment after treatment is also important. Persistent memory, attention, or executive difficulties may justify a more detailed neuropsychological evaluation and targeted support. Overall, cognitive impairment is common and often improves after treatment. However, recovery may remain incomplete and cannot be inferred from radiological resolution alone.

### 3.2. Gait Phenotypes, Mobility, and Balance

Gait disturbance is one of the most common clinical features of cSDH. In the Swiss population-based multicenter cohort, gait disturbance or falls were reported in 58.6% of surgically treated patients. It was the most frequent presenting symptom [9]. Clinically, gait changes may comprise higher-level/frontal gait impairment (gait initiation failure, short shuffling steps, reduced foot clearance, and difficulty turning), hemiparetic gait (asymmetric step length and focal weakness), cerebellar/ataxic-appearing gait (variable step placement and wide base), or parkinsonian features (bradykinesia and freezing). In occasional cases, patients may develop a short-stepped or shuffling gait with difficulty initiating movement, resembling a frontal or parkinsonian gait disorder [27,28]. Gait impairment may coexist with hemiparesis or other focal motor deficits, although these manifestations should be assessed separately [29].

Sundström et al. [12] provided one of the few objective assessments of postural control in cSDH. They measured trunk sway during standing and walking in 22 patients and 58 healthy older adults. Patients with cSDH performed worse during every standing task. During walking, they had a larger sway angle and moved more slowly. Only 18% completed all tasks without losing balance. Early postoperative testing suggested partial improvement in postural stability. However, only five patients were available for this assessment. The study also found no clear relationship between balance impairment and hematoma size, midline shift, side, or patient age [12]. A small radiological lesion or limited midline shift should therefore not be assumed to indicate a low fall risk.

Gait assessment should distinguish static standing balance, dynamic balance during turns and transfers, habitual gait speed, and the capacity to perform real-world activities safely. Fall precautions are particularly important during the early hospital period. Current multidisciplinary guidelines for cSDH include rehabilitation and recovery as distinct parts of the patient pathway [16]. This reflects a broader shift from treating the hematoma alone to supporting the patient’s return to safe daily function [30].

A structured bedside examination should document gait initiation, stride length and asymmetry, turning, freezing, arm swing, muscle tone, resting tremor, and associated focal neurological signs. In patients with established Parkinson’s disease, changes relative to the usual levodopa-responsive motor state and medication timing should be recorded; an acute deterioration should not automatically be attributed to Parkinson’s disease progression. Instrumented gait analysis, gait speed over a measured distance, and dual-task walking may reveal deficits not apparent on standard observation, but their measurement properties and thresholds have not been established specifically for cSDH. These measures are therefore supplementary rather than routine mandatory tests.

### 3.3. Why These Symptoms Occur

The mechanisms behind cognitive and gait impairment in cSDH are not fully understood. A simple mass-effect model is probably incomplete. The hematoma can deform the cerebral hemispheres and displace deeper structures. Mechanical distortion may plausibly affect frontal-subcortical and thalamocortical pathways relevant to attention, motor planning, and postural control; direct network-level evidence in cSDH remains limited [31].

Perfusion studies support the involvement of both cortical and deep brain regions. Inao et al. [32] found that mental changes were associated with reduced blood flow in the ipsilateral frontal cortex and both thalami. The degree of local perfusion reduction was also related to measures of brain displacement. These findings suggest that symptoms may reflect disruption of distributed neural networks rather than dysfunction of the compressed cortex alone [32].

Tanaka et al. [33] examined cerebral blood flow and intracranial pressure before and after surgery in 15 patients. Cerebral blood flow was markedly reduced before treatment. However, all patients improved clinically one day after surgery, while blood flow and intracranial pressure remained abnormal. Perfusion improved more slowly during the following weeks. The authors proposed that early symptoms were driven mainly by mechanical distortion of central structures, particularly the thalamus. They also suggested that this distortion could suppress the function of connected but anatomically distant regions [33].

Other studies indicate that cerebral perfusion still contributes to the clinical picture. Kaplan et al. [34] examined ten patients with unilateral cSDH using transcranial Doppler ultrasonography. Before surgery, blood flow in the ipsilateral middle cerebral artery was reduced, while the pulsatility index was increased. Both measures improved by the third postoperative day, together with consciousness and motor function [34]. The study was very small, and its findings cannot establish a direct causal pathway. When considered alongside the results of Tanaka et al., it suggests that perfusion changes are relevant but are unlikely to be the only cause of neurological dysfunction.

**Figure 1:**
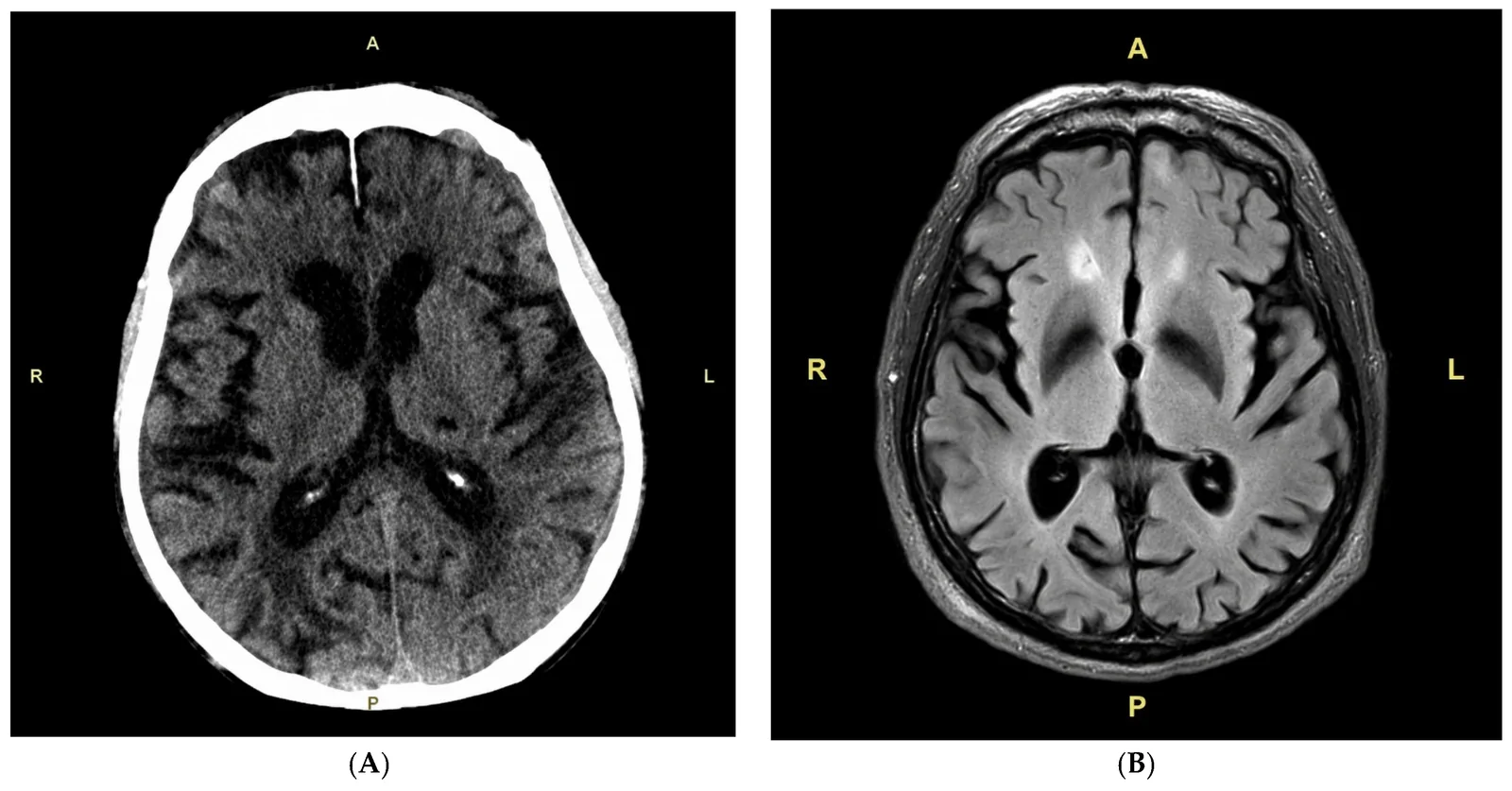
(**A**) Axial non-contrast CT image of the brain showing cortical and subcortical atrophy with widening of the intracranial cerebrospinal fluid spaces, consistent with cerebral atrophy. (**B**) Axial T2-weighted FLAIR image of the brain acquired on a 3-T MRI scanner, showing cortical and subcortical atrophy with enlargement of the intracranial cerebrospinal fluid spaces, consistent with cerebral atrophy. Neither panel depicts a subdural hematoma; both illustrate the predisposing anatomical context rather than cSDH itself. Own material. A—anterior; L—left; P—posterior; R—right.

Age-related changes may further influence the clinical response to cSDH. Brain atrophy increases the available subdural space and contributes to hematoma formation (Figure 1). In older adults, frailty may complicate clinical assessment and is associated with less favorable functional outcomes [35]. Weigel et al. [36] emphasized that aging affects tissue repair, inflammatory regulation, vascular responses, and cellular regeneration. These factors may reduce the brain’s ability to compensate for gradual compression. They may also limit recovery after the hematoma has been treated [35,36,37].

The combined effects of mechanical deformation, network disruption, altered perfusion, and reduced neurological reserve offer a plausible explanation for the wide clinical variation seen in cSDH. They may also explain why cognitive slowing and gait instability often occur together. Importantly, symptom severity does not always correspond closely to hematoma thickness or midline shift. Clinical assessment must therefore remain central, even when radiological findings appear modest.

## 4. Clinical Recognition and Differential Diagnosis

Clinical recognition is often more difficult than the radiological diagnosis of cSDH. Once neuroimaging is performed, the hematoma is usually easy to identify. The main challenge is deciding which patients should be scanned. This can be difficult in older adults, who often have pre-existing cognitive impairment, gait problems, frailty, or several comorbidities. The diagnosis therefore depends on recognizing a new change from the patient’s usual level of function [16,38].

### 4.1. When to Suspect cSDH

cSDH should be considered when neurological or functional symptoms worsen over several days or weeks. Common warning signs include headache, speech disturbance, unsteadiness, recurrent falls, focal weakness, confusion, and reduced consciousness [39]. The presentation is usually gradual, but sudden or transient focal neurological deficits may also occur [40]. Seizures are less common but may occasionally be present at the time of diagnosis [16,41].

The combination of cognitive decline and gait disturbance is particularly important. Neither symptom is specific when considered alone. However, their recent onset or simultaneous progression should raise suspicion of a structural brain disorder. New loss of independence, reduced walking ability, repeated falls, or increasing need for help may be the first signs noticed by relatives or caregivers. The patient may not describe the symptoms clearly and may not remember a preceding head injury. A collateral history is therefore often essential [16].

cSDH is not diagnosed only in neurosurgical departments. Many patients first present to primary care, emergency medicine, geriatrics, internal medicine, or stroke services. In a prospective study of patients aged 75 years or older admitted to a geriatric department, falls and progressive neurological deficits were the most common presenting features. A history of head trauma was documented in only 37% of patients. Moreover, only 38% of the available CT requests mentioned suspected cSDH or another space-occupying lesion. Most scans had been requested because of possible stroke or rapidly increasing confusion [42].

The absence of known trauma should not exclude the diagnosis. Minor falls may have occurred several weeks earlier and may no longer be remembered. This is especially likely in patients with baseline cognitive impairment. Suspicion should be higher in people receiving anticoagulant or antiplatelet therapy, as well as in those with coagulopathy, alcohol misuse, recurrent falls, or marked frailty [16,38].

Cognitive symptoms should not be dismissed as normal aging. In a cohort of 1307 patients, cognitive complaints at presentation were independently associated with a worse functional outcome [26]. They may therefore identify a particularly vulnerable group. At the same time, isolated confusion is not specific for cSDH. Infection, metabolic disturbance, medication effects, and other causes of delirium should be investigated in parallel [16,26].

A practical rule is to suspect cSDH when an older patient develops a new and otherwise unexplained change in cognition, gait, balance, speech, consciousness, or focal neurological function. Concern should increase when more than one of these features appears together or progresses over time.

The most relevant clinical features that should raise suspicion of cSDH in an older adult are summarized in Table 1.

### 4.2. Differential Diagnosis

The symptoms of cSDH overlap with many common disorders of older age. No single clinical feature can reliably confirm or exclude the diagnosis. The pattern and time course of deterioration are helpful, but neuroimaging is usually required to distinguish cSDH from its main alternatives [16,25].

Delirium is one of the most frequent competing diagnoses. It may cause confusion, reduced attention, altered behavior, and fluctuating consciousness [43]. cSDH can produce a similar picture, particularly in frail patients. The two conditions may also coexist [16,38]. For example, an older patient with cSDH may develop infection, dehydration, or an electrolyte disturbance that further worsens cognition. Finding a possible cause of delirium should not automatically end the diagnostic process when neurological symptoms, falls, headache, or progressive decline are also present.

Neurodegenerative dementia usually develops over months or years. In contrast, deterioration related to cSDH is often more recent and may progress over days or weeks. Psychomotor slowing, memory loss, apathy, and disorientation can nevertheless resemble dementia [44]. Schebesch et al. [25] emphasized that this presentation may delay neuroimaging, especially when symptoms are initially attributed to a neurodegenerative disorder. A previous diagnosis of dementia does not protect against cSDH. The key question is whether the patient has changed from their usual cognitive and functional baseline; when no formal premorbid testing exists, informant history and earlier care records provide only an approximate baseline and limit causal attribution.

An illustrative case described a 91-year-old woman with new confusion, impaired memory, and gait dyspraxia. She had no known history of trauma and no clear focal deficit. CT eventually revealed a large subdural hematoma with marked brain displacement. Her mental and functional status improved after evacuation [45]. This report should not be used to estimate the frequency of such presentations. It does, however, show how cSDH may be missed when cognitive symptoms dominate the clinical picture.

Stroke and transient ischemic attack are also important alternatives. Both may cause focal weakness, aphasia, sensory loss, or sudden gait difficulty [46]. cSDH more often follows a gradual course, but it can occasionally produce sudden or transient symptoms. A stroke-like presentation should therefore not be assumed to represent an ischemic event without brain imaging. The possibility of cSDH is particularly relevant when symptoms fluctuate, follow repeated falls, or occur together with cognitive decline [16,42].

Normal-pressure hydrocephalus may resemble cSDH because both conditions can present with gait impairment and cognitive decline. Urinary symptoms and ventriculomegaly support normal-pressure hydrocephalus, but clinical features may overlap [47]. Parkinsonian disorders should also be considered when short steps, gait initiation failure, rigidity, freezing, or a resting tremor are present [48]. However, rapidly progressive gait freezing may occasionally have a structural cause. In one case, freezing of gait resolved after evacuation of a left-sided cSDH [49]. As this was a single case, it shows what is possible rather than what is typical.

Secondary parkinsonism associated with cSDH has been described primarily in case reports and small clinical series, sometimes with improvement after hematoma evacuation [50]; such observations do not establish its frequency or distinguish it reliably from coexisting idiopathic Parkinson’s disease. A pre-existing ventriculoperitoneal shunt for idiopathic normal-pressure hydrocephalus is relevant because overdrainage can predispose to subdural collections; a patient with new imbalance after shunting warrants imaging and evaluation of the shunt setting [51]. Subdural hematoma has also been reported after deep brain stimulation procedures, although postoperative gait deterioration has multiple potential causes [52]. Intracranial hypotension, particularly in bilateral collections or with orthostatic headache or diffuse pachymeningeal enhancement, should be considered as an underlying contributor rather than assumed to be a separate diagnosis [53].

Depression may cause reduced activity, poor concentration, apathy, and subjective memory problems [44]. Sedatives, opioids, anticholinergic drugs, antipsychotics, and some cardiovascular medications may also affect cognition and balance [54]. Frailty and physical deconditioning can further reduce walking ability. These explanations are common in older adults, but they should not be accepted without reconsideration when deterioration is new, progressive, or accompanied by focal signs, headache, drowsiness, or repeated falls [16].

The differential diagnosis should therefore remain broad. In practice, more than one disorder may be present. A patient may have dementia, frailty, infection, and cSDH at the same time. The purpose of assessment is not simply to choose one diagnosis. It is to identify all potentially treatable contributors to the patient’s decline.

### 4.3. Clinical and Imaging Assessment

#### 4.3.1. Clinical Assessment

Assessment should begin with the time course of symptoms. The clinician should establish when the change started and whether it has progressed, fluctuated, or occurred suddenly. Previous cognitive and physical function should be documented. Information from relatives or caregivers is often more reliable than the patient’s account. The history should cover falls, head trauma, headache, seizures, alcohol use, antithrombotic treatment, bleeding disorders, and recent medication changes [16,55].

The examination should include the Glasgow Coma Scale (GCS) and a standard neurological assessment. Particular attention should be paid to speech, facial asymmetry, limb strength, coordination, sensation, and visual fields. Cognition, attention, gait, balance, transfers, and ability to stand should also be assessed. A normal limb-strength examination does not exclude clinically important gait or cognitive impairment. Frailty, premorbid independence, comorbidities, and the patient’s treatment preferences should be recorded because they influence later decisions [16,56].

Current guidelines recommend a full blood count, coagulation tests, fibrinogen, renal function, electrolytes, and electrocardiography after cSDH is diagnosed. Additional tests should be guided by the clinical picture. They may be needed to identify infection, metabolic disturbance, or another contributor to confusion. Patients with confirmed cSDH should be referred urgently for neurosurgical review. The referral should describe the symptoms, their trajectory, the GCS, previous function, frailty, antithrombotic therapy, and relevant treatment wishes [16].

#### 4.3.2. Imaging Assessment

**Figure 2:**
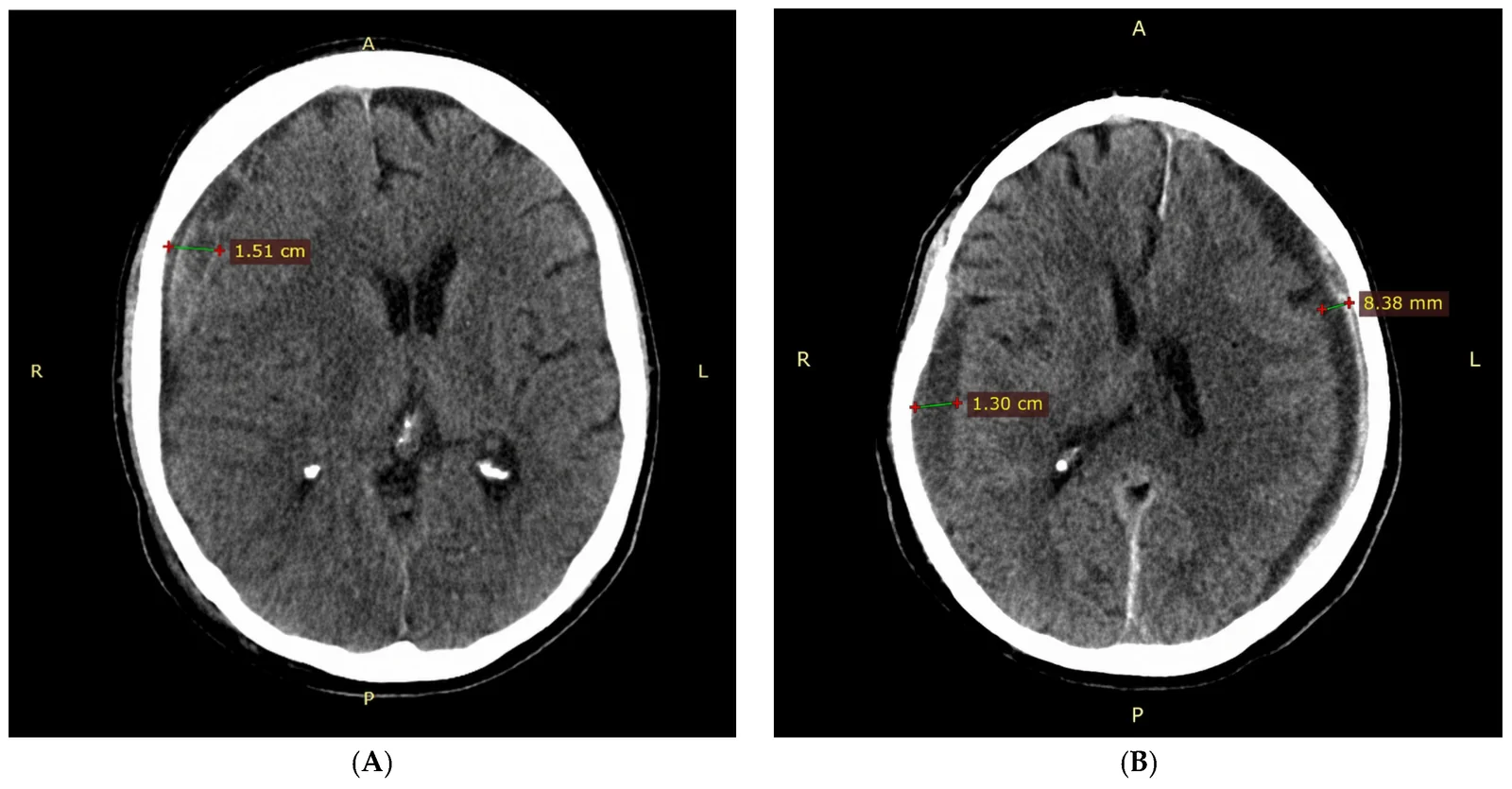
(**A**) Axial non-contrast CT image of the brain showing a right-sided subdural hematoma causing marked compression of the brain parenchyma, with the maximum visible thickness measurement indicated and ipsilateral sulcal effacement. (**B**) Axial non-contrast CT image of the brain showing bilateral subdural hematomas causing marked bilateral compression of the brain parenchyma, with bilateral thickness measurements and compression of the adjacent cerebral hemispheres; midline displacement can be modest despite bilateral mass effect. Own material. A—anterior; L—left; P—posterior; R—right.

Non-contrast computed tomography (CT) is the first-line imaging test when cSDH is suspected. It is fast, widely available, and usually sufficient to establish the diagnosis. A typical cSDH appears as a crescent-shaped extra-axial collection (Figure 2). It may be hypodense, isodense, hyperdense, or mixed in attenuation. Mixed-density areas may reflect blood of different ages or recent bleeding into a chronic collection [16,57].

CT assessment should describe the side and extent of the hematoma, its maximum thickness, and the degree of midline shift. The report should also address sulcal effacement, ventricular compression, basal cisterns, and signs of herniation [12,16,57]. Bilateral hematomas may produce substantial brain compression without a large midline shift. Clinical severity should therefore not be inferred from midline shift alone [58].

The internal structure of the collection may also be relevant. Membranes, septations, fluid levels, and mixed-density components can reflect different stages of hematoma organization. These findings may influence treatment planning and may be associated with persistence or recurrence [57,59]. However, radiological classifications should support rather than replace clinical assessment.

**Figure 3:**
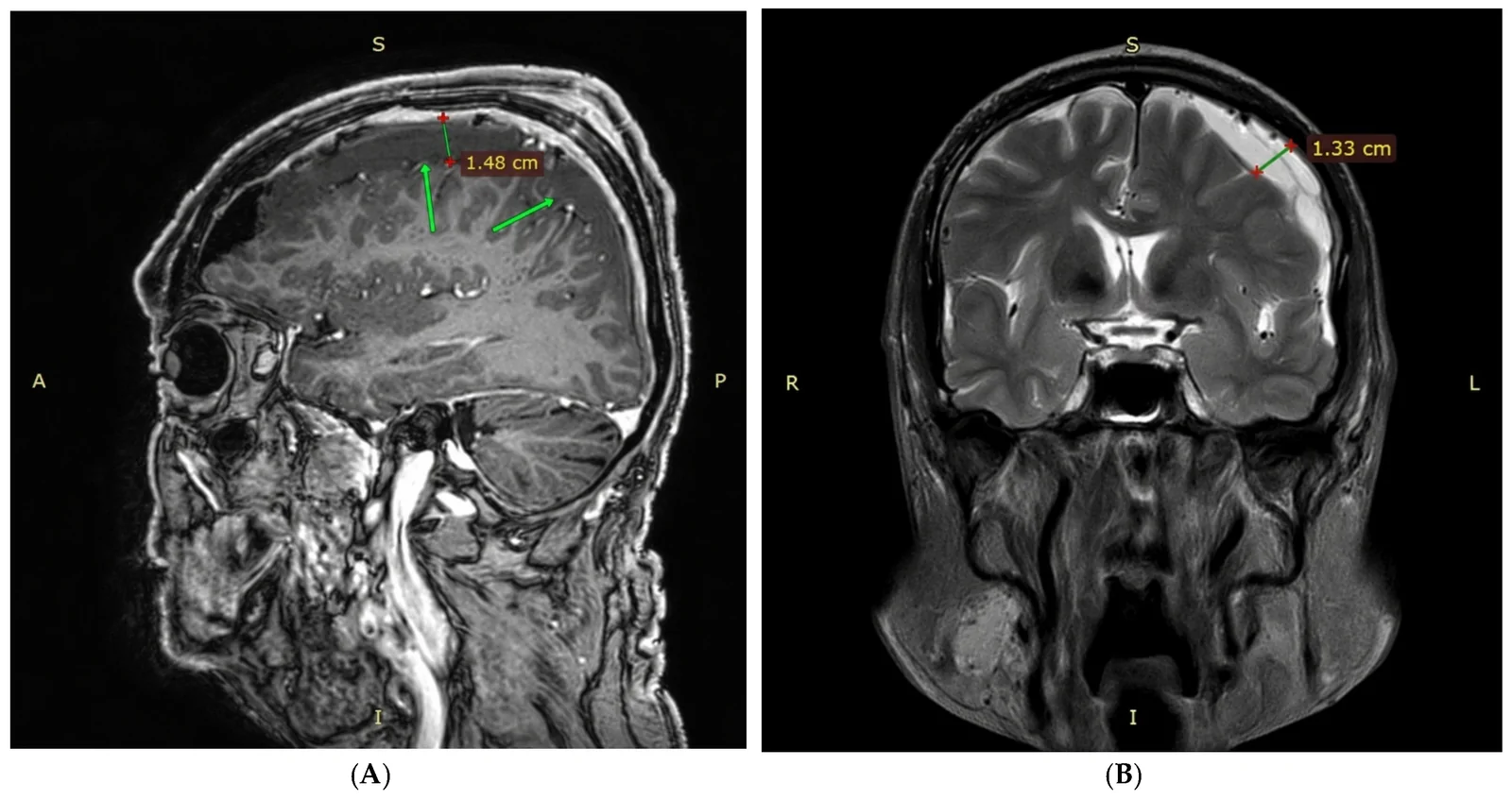
(**A**) Sagittal T1-weighted contrast-enhanced 3-T MRI image of the brain showing an isointense subdural hematoma over the right cerebral hemisphere, indicated by arrows. (**B**) Coronal T2-weighted 3-T MRI image of the brain showing a hyperintense subdural hematoma over the left cerebral hemisphere, with the hematoma thickness indicated. Own material. A—Anterior; I—Inferior; L—Left; P—Posterior; R—Right; S—Superior.

Magnetic resonance imaging (MRI) is not required in most typical cases because non-contrast CT is usually sufficient to establish the diagnosis. MRI may nevertheless be useful when CT findings are equivocal, when an isodense collection is difficult to distinguish from the adjacent brain, or when an alternative diagnosis is suspected [60]. MRI is more sensitive than CT for demonstrating the internal architecture of cSDH, including septations and intrahematomal membranes [60,61]. Contrast-enhanced MRI can additionally demonstrate enhancement of the outer and inner membranes and may help characterize more organized hematoma components (Figure 3) [61]. Recent studies have also explored contrast-enhanced MRI and dual-energy CT for membrane characterization and the identification of imaging markers potentially associated with recurrence. These techniques remain complementary investigations rather than routine first-line imaging methods [57,59].

Bilateral cSDH may be associated with intracranial hypotension, including cerebrospinal fluid leakage or excessive shunt drainage. When the history or imaging raises this possibility, specialist neurosurgical or neuroradiological assessment is appropriate [16].

Imaging findings should always be interpreted together with the clinical course. Recent consensus recommendations support surgery when the hematoma produces significant mass effect that threatens life or mobility. Conservative care may be considered in patients with mild symptoms and limited mass effect, provided that close clinical and radiological follow-up is available. Worsening symptoms require renewed assessment and often a change in management [62].

The aim of assessment is therefore not only to confirm the presence of a hematoma. It is also to determine whether the radiological lesion explains the patient’s decline, whether urgent intervention is needed, and which additional conditions may affect recovery.

## 5. Treatment

Treatment of cSDH should address more than the hematoma itself. The immediate aim is to relieve brain compression and prevent further neurological deterioration [63]. However, treatment success should not be defined solely by hematoma evacuation or the prevention of recurrence. It should also include recovery of cognition, mobility, functional independence, and quality of life. The treatment strategy should therefore be individualized according to symptom severity, radiological mass effect, frailty, comorbidity burden, antithrombotic therapy, and the patient’s premorbid level of function [62,64,65].

### 5.1. Surgical Treatment

Surgical evacuation remains the standard treatment for patients with symptomatic cSDH and clinically relevant mass effect. Burr-hole evacuation with postoperative drainage is the most widely used approach. It provides rapid decompression while limiting operative trauma. Twist-drill drainage may be suitable for selected patients with a predominantly liquid hematoma [66]. Craniotomy is usually reserved for organized, multiloculated, or recurrent collections that cannot be adequately treated through burr holes [64,67,68]. Earlier systematic reviews likewise support burr-hole or twist-drill drainage for most liquefied collections, while reserving craniotomy for organized or septated hematomas [69,70].

Age alone should not be treated as a contraindication to surgery. A systematic review focused on older adults found that surgical treatment generally produced favorable neurological outcomes. Rates of recurrence, reoperation, and overall complications were similar to those reported in younger patients. Some studies showed higher mortality in older adults, but this appeared to reflect frailty and comorbidity rather than the surgical procedure alone [64]. Clinical decisions should therefore consider biological vulnerability and treatment goals rather than chronological age in isolation.

The choice of surgical technique should remain individualized. A broad component network meta-analysis of 455 studies and more than 100,000 cases found that postoperative drainage reduced recurrence by almost half [67]. For recurrence prevention, the presence of a drain appears more consistently supported than the superiority of a single burr-hole number, irrigation approach, or drain position [67,71]. A more recent network meta-analysis suggested that some subgaleal and irrigating drainage strategies may have advantages [72]. However, the available comparisons remain heterogeneous, and no single drain position can yet be considered universally superior. Randomized evidence also suggests that a subperiosteal drain can reduce drain-related complications, whereas DRAIN TIME 2 supports 24 h of drainage when a fixed-duration protocol is used [73,74].

Surgery also requires careful perioperative planning. Antithrombotic therapy and coagulation abnormalities should be reviewed before treatment, with perioperative interruption and postoperative resumption individualized according to the risks of recurrent bleeding and thromboembolism [75]. Frailty, nutritional status, baseline cognition, comorbidities, and premorbid mobility should also be documented because they may influence functional recovery and discharge needs [76]. Postoperative care should include neurological observation, prevention of medical complications, appropriate management of antithrombotic therapy, and early planning for mobilization and discharge [16,64]. A technically successful operation may still be followed by poor recovery when frailty, infection, immobility, or pre-existing cognitive impairment are not addressed.

Pharmacological treatment should not be assumed to provide an equivalent alternative to surgery in symptomatic patients. In the randomized DECSA trial, dexamethasone did not establish noninferiority to burr-hole drainage for three-month functional outcomes and was associated with a higher complication burden. Complications were more frequent with dexamethasone, and more than half of the patients assigned to dexamethasone later required surgery. The trial was stopped early because of concerns about safety and outcomes [77]. These findings argue against using dexamethasone as a routine substitute for surgical evacuation in patients who are suitable for surgery. These results are consistent with the Dex-CSDH trial, in which dexamethasone was associated with worse functional outcomes and more adverse events despite fewer repeat operations [78].

Atorvastatin has shown potential as a conservative treatment in selected patients with mild-to-moderate cSDH. In the phase II ATOCH trial, atorvastatin produced greater hematoma-volume reduction than placebo in selected patients with mild-to-moderate cSDH; the effects on patient-centered function and the applicability to other populations remain uncertain. However, the evidence is population-specific and does not justify replacing decompression in patients with clinically significant mass effect [79].

Tranexamic acid has also been investigated as a medical approach to hematoma resolution, but the 2024 multidisciplinary guideline recommends against its routine use for cSDH outside research because evidence is insufficient [16]. Some more recent interventional guidance allows carefully selected use with explicit attention to thromboembolic risk; these positions reflect evolving and uneven evidence, not established functional efficacy [80]. Timing of antiplatelet and anticoagulant resumption after evacuation must weigh the indication for treatment and thromboembolic risk against stability and recurrence risk; a 2026 systematic review of seven studies (3195 patients) did not demonstrate a significant recurrence difference between early and delayed resumption, but definitions and estimates were heterogeneous [81]. Evidence is likewise insufficient to recommend routine antiseizure-drug prophylaxis after uncomplicated cSDH evacuation in patients without seizures, and treatment decisions should consider seizure history, cortical injury, and perioperative events rather than assuming a universal preventive benefit [41]. None of these drug strategies has established an independent improvement in cognition or gait.

The absence of recurrence should be distinguished from the patient’s functional status. Improvement in cognition and gait may occur after decompression, but it is neither immediate nor complete in every patient. Continued assessment is therefore needed after surgery, especially when memory problems, poor balance, or loss of independence persist.

### 5.2. Middle Meningeal Artery Embolization

**Figure 4:**
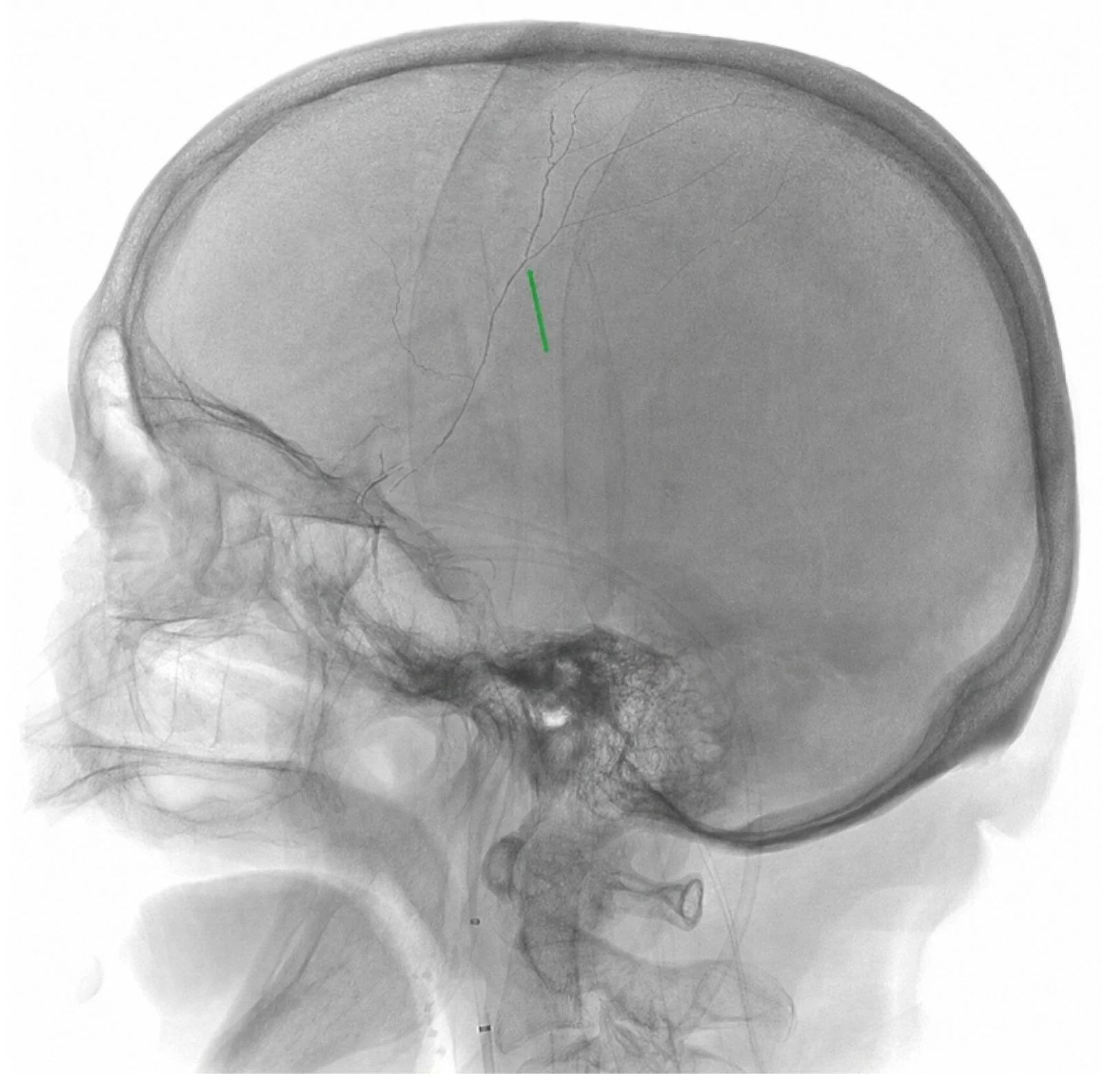
Lateral skull radiograph showing embolic material within the lumen of the left middle meningeal artery (25% n-butyl cyanoacrylate), indicated by the green arrow. Own material.

Middle meningeal artery embolization (MMAE) occludes distal branches of the middle meningeal artery that supply the pathological neovasculature of the outer hematoma membrane. By reducing blood flow to this fragile capillary network, the procedure aims to interrupt the cycle of inflammation, angiogenesis, vascular leakage, and repeated microhemorrhage that contributes to hematoma persistence and recurrence [82]. Unlike surgical evacuation, MMAE does not immediately remove the subdural collection or rapidly relieve clinically significant mass effect. Radiological resolution usually occurs gradually over the following weeks or months [83]. MMAE should therefore be considered an adjunctive or alternative treatment in appropriately selected patients rather than a universal replacement for surgical decompression [57]. Representative angiographic findings and the post-embolization distribution of the embolic agent are shown in Figure 4.

The EMBOLISE trial evaluated MMAE as an adjunct to surgery in patients with symptomatic subacute or chronic subdural hematoma. Recurrence or progression requiring repeat surgery within 90 days occurred in 8/197 patients (4.1%) treated with adjunctive MMAE and surgery versus 23/203 (11.3%) after surgery alone (absolute difference, 7.2 percentage points; relative risk, 0.36; 95% CI, 0.11–0.80; *p* = 0.008). Neurological functional deterioration was similar between groups. However, serious procedure-related adverse events occurred in 2% of embolized patients, including two disabling strokes [84]. The trial therefore demonstrated a meaningful reduction in reoperation, but it also confirmed that MMAE is not without procedural risk.

The results of MAGIC-MT and STEM provide a broader view. MAGIC-MT included 722 patients, most of whom underwent burr-hole drainage. Symptomatic recurrence or progression at 90 days occurred in 24/360 patients (6.7%) assigned to MMAE and 36/362 (9.9%) receiving usual care (risk difference, −3.3 percentage points; 95% CI, −7.4 to 0.8; *p* = 0.10); serious adverse events were less frequent after MMAE [85]. In STEM, the 180-day composite treatment-failure endpoint occurred in 19/120 (16%) patients assigned to MMAE versus 47/129 (36%) controls (odds ratio, 0.36; 95% CI, 0.20–0.66; *p* = 0.001). The STEM endpoint was broader and included residual or recurrent hematoma, surgical rescue, disabling vascular events, and neurological death. Short-term rates of disabling stroke or death were similar between groups [86].

These trials used different embolic agents, populations, concomitant surgical pathways, follow-up periods, and non-equivalent primary endpoints; pooled estimates require caution. A 2025 synthesis of six randomized trials suggested lower recurrence after MMAE, but individual trials and definitions differed. It did not show a clear improvement in global functional outcome, independent ambulation, or mortality [87]. A newer 2026 meta-analysis of four large multicenter trials found the strongest effect among patients initially managed without surgery. In that group, MMAE substantially reduced treatment failure and the need for later surgical rescue. Its benefit as a routine surgical adjunct was smaller, although still statistically significant in the pooled analysis [88]. These findings support a selective approach based on the initial treatment pathway and individual recurrence risk.

The multicenter MEMBRANE randomized trial [89] evaluated n-BCA MMAE plus standard care versus standard care alone in 376 randomized patients. At six months, the primary composite event was reported in 17/146 (11.6%) versus 29/131 (22.1%) among patients with evaluable endpoint data (common odds ratio, 0.53; 90% CI, 0.31–0.91; *p* = 0.04). These denominators differ from the randomized totals (188 per arm) because endpoint data were incomplete; the trial used a 90%, not 95%, confidence interval. The primary composite included residual or reaccumulated hematoma or additional surgery and must not be interpreted as equivalent to EMBOLISE reoperation or MAGIC-MT symptomatic recurrence. Reported three-month global functional noninferiority does not establish improvement in cognition, gait, or participation.

The available evidence also reveals an important limitation. Modern MMAE trials have focused mainly on recurrence, radiological progression, reoperation, composite treatment failure, mortality, and the modified Rankin Scale (mRS). Detailed cognitive testing, objective gait assessment, and patient-reported recovery have rarely been primary or adequately powered outcomes; MAGIC-MT reported quality-of-life measures as secondary outcomes, but these should not be conflated with a demonstrated cognitive or gait benefit. It therefore remains unclear whether the reduction in radiological recurrence translates into better memory, safer walking, greater independence, or improved quality of life. Future trials should include these outcomes, particularly in older adults whose main treatment goal may be functional recovery rather than radiological resolution alone.

### 5.3. Rehabilitation

Recovery after cSDH treatment may be limited by weakness, poor balance, cognitive impairment, fear of falling, and medical deconditioning. These problems may persist even after satisfactory hematoma evacuation. Rehabilitation should therefore begin during the acute hospital stay and continue after discharge when needed. Assessment should include mobility, transfers, standing balance, gait safety, cognition, daily activities, and the patient’s home environment [16,90].

Carlisi et al. [90] evaluated a postoperative rehabilitation program in 35 older patients treated with burr-hole drainage. Daily physiotherapy focused on standing and walking. Trunk control, standing balance, neurological status, and general function improved before discharge. Approximately three-quarters of previously ambulatory patients retained the ability to walk, but fewer than half were discharged directly home. The study had no control group, so it cannot establish the independent effect of rehabilitation. It nevertheless shows that residual disability often remains important after successful surgery.

The GET-UP randomized trial provided stronger evidence for early mobilization. Patients assigned to the early-mobilization group began head elevation within 12 h after surgery and progressed toward sitting, standing, and walking as tolerated. The comparison group remained on bed rest for 48 h. Medical complications occurred in 20/104 (19.2%) early-mobilization patients versus 36/104 (34.6%) assigned to 48 h bed rest (*p* = 0.012). Early mobilization did not significantly increase surgical recurrence [91]. These findings suggest that prolonged routine bed rest may expose older patients to avoidable harm without providing clear protection against hematoma recurrence.

At one year, a favorable Glasgow Outcome Scale–Extended (GOSE) score (≥5) was observed in 78/102 patients (76.5%) assigned to early mobilization compared with 59/101 (58.4%) after bed rest (*p* = 0.006; adjusted odds ratio, 2.006; 95% CI, 1.076–3.739). Surgical recurrence was similar (6.9% vs. 5.9%; *p* = 0.788), and the observed mortality difference (15.7% vs. 24.8%; *p* = 0.108) was not statistically significant. These findings are clinically relevant because they extend beyond the early postoperative period. However, GET-UP was a single-center, open-label trial. The intervention also varied according to what each patient could tolerate [92]. Its results should therefore be confirmed in other healthcare systems and patient populations.

A 2026 systematic review identified only four comparative studies, comprising 622 patients. No included study established a significant increase in recurrence with early mobilization. Some studies reported fewer medical complications, shorter hospital stays, or better functional recovery. In contrast to the significant primary complication result in GET-UP, pooled analyses of the four studies did not establish statistically significant differences in either recurrence or medical complications. Definitions of early mobilization, treatment protocols, and outcome measures varied considerably [93]. The evidence is therefore encouraging but still limited.

The apparent discrepancy reflects differences in the timing and definition of mobilization (ambulation within 48 h versus prescribed 48 h or longer bed rest), clinical stability criteria, comparator protocols, and outcome ascertainment. A pooled null result does not prove equivalence and does not invalidate an individual randomized result; equally, it does not justify a universal postoperative mobilization schedule.

Current guidance supports early, individualized mobilization rather than mandatory prolonged bed rest [16]. The plan should reflect neurological status, cardiovascular stability, drain management, premorbid function, and fall risk. Enhanced recovery pathways may help coordinate mobilization, thromboprophylaxis, medication review, nutrition, and discharge planning, although it remains difficult to separate the effect of each individual component [30].

Rehabilitation should not end when the patient can walk along a hospital corridor. Some patients continue to have reduced endurance, impaired balance, memory problems, or difficulty managing medications and daily activities. A safe discharge plan may require outpatient physiotherapy, occupational therapy, cognitive assessment, home adaptations, or temporary inpatient rehabilitation. Treatment should ultimately aim to restore meaningful function, not only to remove the hematoma.

#### Distinguishing Mobilization, Physiotherapy, and Therapeutic Exercise

Early mobilization refers to positioning, sitting, standing, transfers, and ambulation initiated during the acute postoperative period, primarily to prevent the harms of immobility. Physiotherapy involves clinician-directed assessment and interventions for an identified deficit, for example assisted gait practice, balance retraining, and transfer practice. A structured therapeutic exercise program is more specific: it should define modality, session duration, frequency, intensity, progression, supervision, safety stopping rules, and adherence. GET-UP tested an early mobility protocol, not a standardized resistance, aerobic, or balance exercise prescription [91]. Carlisi et al. [90] described daily therapist-supervised standing and walking practice, but did not establish a validated exercise dose for cSDH. In medically stable patients, a practical individualized approach may progress from supervised transfers and short assisted walks toward gait endurance, task-specific balance exercises, and strengthening according to fatigue, orthostatic tolerance, cognition, fall risk, and neurological findings. These are pragmatic extrapolations from geriatric neurorehabilitation, not trial-proven cSDH dose recommendations. No specific cSDH frequency, intensity, session duration, or progression algorithm can currently be endorsed, and prospective dose-finding and adherence studies are needed.

Selected recovery studies and pivotal randomized treatment trials are compared in Table 2 to separate study design, clinical endpoints, follow-up, and important limitations.

## 6. Recovery After Treatment

Recovery after treatment for cSDH is a multidimensional process. Relief of brain compression may lead to rapid neurological improvement. Recovery of cognition, balance, independence, and social participation may take longer. In some patients, these functions do not return fully to their previous level [15]. Because anatomical and neurological endpoints do not capture all recovery domains, clinical testing should track cognition, gait safety, and independence separately.

Because recovery after cSDH is multidimensional, the main patient-centered domains that should be assessed after treatment are summarized in Table 3.

### 6.1. Cognitive Recovery

Cognitive recovery can begin soon after surgical evacuation of the hematoma. Improvements in global cognitive screening scores have been observed within the first 24–48 h after surgery [94]. This early change may reflect relief of cerebral compression and mechanical distortion of cortical and subcortical networks. In the prospective study by Gill et al. [95], cognitive function was assessed before surgery and again 24 h after burr-hole evacuation. The mean Mini-Mental State Examination score increased from approximately 23 to 27. Most patients who met the selected threshold for cognitive impairment before surgery scored above that threshold during the early postoperative assessment. This rapid change supports the concept that part of the cognitive syndrome is directly related to the hematoma. However, the result should be interpreted cautiously. The study included only 30 patients and had no control group. The same screening test was repeated after a very short interval. Practice effects, improved alertness, relief of headache, and changes in the hospital environment may therefore have influenced the result [95]. Early improvement does not necessarily mean that memory, executive function, and complex daily decision-making have fully recovered.

The systematic review by Blaauw et al. [18] supports early postoperative improvement, but the extent of individual recovery remains uncertain because cognitive instruments, definitions, assessment intervals, and premorbid data differ. The direction of change was consistent with meaningful recovery. Nevertheless, the studies used different tests, definitions, and thresholds. Most postoperative assessments were performed between 24 h and two weeks after treatment. Premorbid cognitive status was also rarely known. The available evidence therefore describes early recovery better than the full cognitive trajectory.

The situation is less reassuring when cognition is assessed after a longer interval. Approximately three months after treatment, 54% of patients in an exploratory study had test scores consistent with cognitive impairment. Their overall cognitive scores remained lower than those of healthy controls, with the clearest difference in verbal memory. Patients with known or suspected dementia had been excluded. This reduces the likelihood that the findings were explained only by established neurodegenerative disease [13]. The study did not include objective preoperative testing, so it could not measure individual improvement from baseline. It still shows that successful treatment does not guarantee normal cognition at three months.

These findings suggest that cognitive recovery follows several possible paths. Some patients improve quickly and remain stable. Others improve only partly or continue to recover over the following months. A third group may have persistent impairment due to pre-existing dementia, cerebral small-vessel disease, frailty, perioperative complications, or incomplete neurological recovery. The hematoma may unmask previously compensated cognitive vulnerability rather than account for all observed deficits. Without premorbid objective testing, untreated control groups, and consistent cognitive instruments, a postsurgical deficit cannot reliably be apportioned among persistent hematoma effects, neurodegeneration, small-vessel disease, or frailty. Acute postoperative delirium, characterized by fluctuating attention or awareness, should be assessed separately from enduring cognitive impairment; reassessment after resolution of acute confounders helps avoid attributing all observed deficits to the hematoma [16,43].

Cognitive assessment should therefore be repeated after treatment. A brief screening test can identify patients who need further evaluation, but it should not be used in isolation. Information from a relative or caregiver can reveal problems with memory, medication management, finances, judgment, and everyday planning that may not be obvious during a short clinical visit. Persistent or functionally important deficits may justify formal neuropsychological assessment and targeted cognitive support.

### 6.2. Gait and Balance Recovery

Gait and postural control may also improve after decompression, but the available evidence is more limited. Sundström et al. [12] observed impaired balance before surgery and tentative postoperative improvement in only five retested patients; this is insufficient to establish an expected recovery trajectory or predictive cutoffs.

In the same small study, postural instability was not clearly correlated with routine radiological metrics [12]. A reassuring radiological appearance should therefore not be used to infer that a patient can walk safely. Balance may remain impaired even when focal weakness has improved. This distinction is clinically important because patients are often at continued risk of falls during the early recovery period.

Motor recovery may be rapid, but it is not universal. In a cohort of 311 surgically treated patients, 68% of those presenting with focal weakness improved by discharge, while 58% achieved complete resolution [96]. Longer symptom duration and older age were associated with less complete motor recovery. Importantly, improvement in strength should not be equated with restoration of balance or safe independent walking.

Small physiological data provide additional reassurance that early postoperative mobilization can be undertaken without marked intracranial pressure elevation. In a small prospective study, intracranial pressure remained within normal limits during supine, head-up, and sitting positions in the first 48 h after surgery, provided that passive drainage was managed appropriately [97]. The cohort included only 11 patients, so these findings should be considered hypothesis-generating.

Objective balance testing may help identify patients whose recovery is slower than expected. In a recent prospective cohort, lower early postoperative Mini-Balance Evaluation Systems Test (Mini-BESTest) scores were associated with longer hospitalization. The Mini-BESTest and the initial neurological deficit both contributed to the observed length of stay. The study analyzed only 32 patients and excluded people unable to complete the test. Its results are preliminary, but they show the potential value of structured balance assessment in rehabilitation and discharge planning [98].

Global scales such as the mRS remain useful, but they do not describe the mechanisms of walking difficulty. A patient may receive the same global score because of weakness, poor balance, cognitive slowing, fear of falling, or reduced endurance. Direct assessment of transfers, standing balance, gait speed, need for assistance, and fall risk provides more practical information about recovery.

### 6.3. Functional Independence and Quality of Life

Functional recovery means more than improvement in strength or consciousness. It includes the ability to live independently, manage daily tasks, return to previous activities, and resume meaningful social roles. These outcomes are especially important to older patients, but they are measured less often than mortality, recurrence, or radiological resolution [19].

Patient-reported quality of life may improve during the first months after surgery. In a cohort of 330 patients, Short Form 36 (SF-36) quality-of-life scores improved by 90 days among assessable participants. However, the ability to complete the assessment independently was itself associated with survival, which highlights the risk of excluding the most vulnerable patients from patient-reported outcome studies [99].

Merrill et al. [100] surveyed 38 patients after surgical evacuation. Among those who had exercised regularly before surgery, approximately 90% returned to exercise within one year. About two-thirds of patients who had previously taken part in recreational sports returned to those activities. Fewer than half participated in physical therapy, while most respondents believed that surgery had improved their quality of life. These findings suggest that a meaningful return to physical activity is possible. However, the study was small, relied on a telephone survey, and included patients who were available and willing to respond. It may therefore overestimate recovery in the wider cSDH population.

Return to a complex activity may require more recovery than is suggested by a basic neurological examination. Katsuki et al. [101] assessed older patients who wished to resume driving after treatment. The evaluation included global cognition, attention, visuospatial function, processing speed, executive function, and memory. Only 19 of 30 patients were eventually considered able to return to driving. Some regained this ability at discharge, while others required repeated assessments for up to four months. Older age, dementia, impaired consciousness at presentation, and poorer cognitive test results were associated with inability to resume driving. This single-centre study cannot define universal driving standards. It does show that symptom relief does not automatically restore the capacity for demanding real-world tasks.

Long-term follow-up reveals further limitations. At a mean of 5.5 years after cSDH, Moffatt et al. [14] found lower functional independence and poorer verbal short-term memory than in matched controls. Survivors also reported lower emotional, psychological, and social well-being, together with more depressive symptoms. The study included 51 survivors, so selection and survivor bias should be considered. Even so, the findings challenge the view that cSDH is fully resolved once the hematoma has been drained.

The recent population-matched study by Petutschnigg et al. [15] extends this observation to approximately ten years after surgery. Survivors reported clinically relevant limitations in cognitive, physical, role, and social functioning. At the same time, emotional functioning and overall quality-of-life scores were broadly similar to population reference values. This apparent contrast is important. People may adapt to persistent limitations and still report an acceptable overall quality of life. A preserved global quality-of-life score should therefore not be interpreted as evidence that all functional domains have returned to normal.

### 6.4. Incomplete or Delayed Recovery

The term “successful treatment” can refer to several different outcomes. It may mean that the hematoma has decreased in size, that focal neurological signs have improved, or that reoperation was avoided. These outcomes do not necessarily mean that cognition, mobility, independence, and previous life roles have been restored [13,14,15,19].

Radiological and functional recovery may occur on different timescales. In a large multicenter cohort treated with MMAE, hematoma thickness decreased progressively over several months, while functional status continued to improve through 6–12 months [102]. These findings suggest that early imaging does not capture the full recovery trajectory.

Recent data show that a notable group of patients does not achieve measurable functional improvement. In a post hoc analysis of the nationwide FINISH trial, approximately 20% had no improvement or deterioration in modified Rankin Scale score at six months after surgery; this secondary post hoc observation does not imply that hematoma size causes functional failure. Pre-existing dementia and the use of a walker before cSDH were independently associated with failure to improve. Smaller hematoma width and less midline shift were also associated with a lower likelihood of functional improvement [103]. This counterintuitive finding should not be interpreted as evidence that smaller hematomas are more harmful. It may indicate that the patient’s disability was less directly caused by mass effect and more strongly related to underlying frailty or neurological disease.

Baseline health appears to influence recovery more consistently than many technical details of surgery [104]. In a cohort of 1226 patients treated with burr-hole drainage, favorable outcome and discharge home were associated with preadmission independence, preserved motor response, lower medication burden, and lower anesthetic risk. Surgical laterality and the number of burr holes were not clearly associated with the measured clinical outcomes. These results support the assessment of premorbid function, multimorbidity, and physiological reserve when discussing prognosis with patients and families [105].

The timing of treatment may also matter. A 2026 multicentre cohort included 1015 surgically treated patients. Those with unfavorable postoperative outcomes had waited longer for surgery than those with favorable outcomes. Each additional day of delay was independently associated with a small increase in the probability of an unfavorable result. The study was retrospective, so residual confounding cannot be excluded [106]. For example, medication management and clinical complexity may have influenced both treatment timing and outcome. The findings nevertheless support avoiding unnecessary delays once surgery has been selected.

Incomplete recovery is particularly common in patients with dementia, frailty, multimorbidity, poor premorbid mobility, or low functional independence [103,105]. Recent age-stratified data also show that poor status on admission and multimorbidity predict unfavorable discharge outcomes across older age groups. Postoperative pneumonia, seizures, bleeding, and altered consciousness add further risk in selected patients [107]. Age by itself is less informative than the patient’s baseline reserve and the complications that occur during treatment [108].

Recovery should therefore be assessed across several related but distinct domains, as summarized in Table 4.

Progress in one area does not guarantee progress in the others [100,101]. Patients and families should be informed that improvement may continue after discharge, but it may also remain incomplete. Follow-up should respond to the patient’s actual difficulties rather than relying only on a postoperative CT scan or a single global outcome score [16].

## 7. Discussion

Recovery after cSDH is shaped by several interacting factors. Some are present before the hematoma develops, while others reflect the severity of brain compression or the course of treatment. Age is relevant, but it rarely acts alone. Premorbid independence, frailty, cognition, nutritional status, neurological severity, postoperative complications, and the opportunity for early mobilization may be equally important [103,107].

The prognostic value of individual variables should be interpreted cautiously. Patients with cSDH are heterogeneous, and outcomes are measured at different time points using different scales. An external validation study of published cSDH prognostic models found that none performed well enough for routine clinical use. Discrimination was poor to modest, and some models systematically overestimated adverse outcomes [109]. Prognostic factors are therefore most useful for identifying needs and planning care. They should not be used to deny treatment or to make definitive predictions for an individual patient.

### 7.1. Patient-Related Factors

Frailty appears to be one of the most important patient-related factors. It reflects reduced physiological reserve and vulnerability to relatively minor stressors. Unlike chronological age, frailty captures differences in mobility, cognition, self-care, comorbidity, and independence between patients of the same age [110,111].

Shimizu et al. [111] evaluated 211 surgically treated patients aged 65 years or older. Frail patients had worse functional outcomes and were much less likely to return home. Their median mRS score at three months was 4, compared with 2 among patients without frailty. However, frailty was not independently associated with outcome after all background variables were entered into the adjusted analysis. Age, nutritional status, and hematoma recurrence remained significant. Importantly, adding the Clinical Frailty Scale substantially improved the predictive performance of the model. Frailty should therefore be seen as a useful summary of patient vulnerability rather than an isolated causal factor.

Long-term data support this interpretation. In a separate analysis of the same 1307-patient cohort also used for cognitive complaints [26], all-cause mortality after cSDH exceeded that of the reference population. Several practical markers of frailty were independently associated with mortality. These included recurrent falls, inability to live independently, difficulty performing self-care, and a greater number of medications. Cognitive problems and psychotropic medication use were also considered as frailty indicators, although they did not all remain independently significant in the final model [110]. This suggests that the prognosis of cSDH is closely linked to the patient’s broader health and functional reserve.

A systematic review published in 2024 included six studies and 4085 patients. Across different frailty instruments, frail patients more often experienced mortality, complications, recurrence, and unfavorable discharge destinations. The studies used different definitions and could not be combined in a formal meta-analysis. Nevertheless, the direction of association was consistent [35]. More recent prospective evidence also found that a higher Clinical Frailty Scale score was associated with poorer neurological recovery and greater mortality after surgery [37]. Taken together, these findings support routine frailty assessment before treatment, while also showing the need for a standardized cSDH-specific approach.

Frailty can also be estimated through routine clinical observations. The Functional Impairment after Burr-Hole Trepanation score, or FIT-score, was developed using age, disorientation, and severe motor deficit at admission. In the original cohort, approximately 30% of patients remained functionally impaired. Age of at least 85 years, disorientation, and marked motor weakness were associated with persistent disability. The model had an area under the receiver operating characteristic curve (AUROC) of 0.77 (95% CI, 0.70–0.83) for discharge impairment and 0.76 (95% CI, 0.70–0.82) for later impairment, with internal-validation values of approximately 0.79 and 0.77; calibration and transportability require further evaluation [112]. It may help identify patients who are likely to need rehabilitation or support after discharge. However, it should not be used as a definitive predictor because it has not been widely externally validated.

Premorbid function may be more informative than age alone. The use of a walking aid, dependence in daily activities, dementia, poor pre-hospital mRS, impaired consciousness, and reduced motor response have all been associated with less favorable recovery [106,112,113]. These variables indicate that some patients have limited reserve before cSDH begins. Even when surgery successfully relieves compression, they may not regain their previous level of independence.

Nutritional status is another relevant and potentially modifiable factor. In a prospective multicenter study, poorer nutritional status was associated with an unfavorable outcome after surgical treatment [114]. Shimizu et al. [111] also found that the Controlling Nutritional Status score was associated with three-month function. Malnutrition may coexist with frailty, cognitive decline, inflammation, and reduced muscle strength. Nutritional screening can therefore add useful information to the assessment of an older patient with cSDH, although evidence that nutritional intervention directly improves outcome remains limited.

The main clinical message is that chronological age should not be considered in isolation. A fit and independent patient in their late eighties may have greater recovery potential than a younger patient with dementia, recurrent falls, poor nutrition, and dependence in daily activities. Assessment should focus on biological vulnerability, premorbid function, and the patient’s own goals.

### 7.2. Hematoma-Related Factors

Radiological severity can contribute to neurological impairment, but its relationship with recovery is not straightforward. Hematoma thickness and volume, midline shift, bilaterality, internal architecture, and the size of the residual postoperative cavity have all been investigated as potential radiological prognostic markers [115]. Their clinical significance varies according to the outcome being assessed and the duration of follow-up. Imaging features associated with recurrence may not necessarily predict long-term functional independence, while some studies have linked greater hematoma thickness and midline shift to unfavorable functional outcomes [115,116].

Bilateral cSDH deserves particular attention. In a retrospective series of 368 surgically treated patients, bilateral hematomas were associated with rapid deterioration and a higher risk of poor outcome related to brain herniation. Recurrence rates were similar between bilateral and unilateral cases, but poor outcomes caused by herniation were more common with bilateral disease. The authors suggested that bilateral hematomas may progress rapidly because compensatory midline displacement is limited [117]. These findings support close observation and timely decompression when bilateral collections are accompanied by worsening symptoms.

However, bilaterality does not consistently predict all longer-term outcomes. In an older cohort described by Miranda et al. [118], hematoma size, midline shift, bilateral involvement, anticoagulant use, and type of treatment were not independently associated with long-term mortality. Neurological condition at admission was the main predictor of in-hospital death. Age and discharge destination were more relevant after hospitalization. This contrast illustrates an important point: bilateral hematomas may increase the risk of acute deterioration, but long-term survival and independence are strongly influenced by the patient’s baseline health.

Hematoma volume and midline shift may also have different clinical meanings. A 2025 study of 293 patients with unilateral cSDH found that greater preoperative hematoma volume was associated with hemiparesis and gait disturbance. Midline shift was more closely associated with impaired consciousness. Hematoma volume, postoperative cavity volume, and CT morphology predicted recurrence, while midline shift did not [119]. These findings suggest that volume reflects the burden of the collection, whereas midline shift may better represent deformation of central brain structures.

Newer data suggest that midline shift may still be relevant to early functional status. A 2026 single-center study of 260 patients found that greater midline shift, older age, and worse pre-hospital mRS were independently associated with poor function at discharge. Hematoma thickness showed a similar numerical trend but did not meet the predefined threshold for statistical significance. The full model had an apparent area under the curve (AUC) of 0.733 and an optimism-corrected AUC of 0.721 (95% CI, 0.652–0.790). Internal calibration was acceptable (Hosmer–Lemeshow *p* = 0.42; Brier score 0.168), but independent external validation is lacking. The authors appropriately described the findings as exploratory and advised against using them as a stand-alone basis for clinical decisions [120].

These observations are not necessarily contradictory. Midline shift may be associated with impaired consciousness and early functional disability, while its relationship with recurrence and long-term mortality is less consistent. Similarly, observed associations of hematoma volume with focal motor symptoms or recurrence do not necessarily imply an independent causal effect on long-term function [119]. These differences emphasize that the prognostic significance of a radiological marker depends on the specific outcome and follow-up period being assessed.

Radiological markers should also be interpreted in the context of brain atrophy. An older patient may tolerate a large collection with limited midline shift because more subdural space is available. Another patient may develop marked symptoms from a smaller lesion because neurological reserve is lower. Bilateral hematomas can cause substantial compression without producing a large shift in the midline [57,120].

Overall, radiological burden may contribute to early clinical impairment. It may also affect the risk of persistence or recurrence. However, long-term recovery appears to depend strongly on premorbid function, frailty, neurological condition, and complications. Imaging should support clinical assessment rather than replace it.

### 7.3. Treatment-Related Factors

Treatment-related factors include surgical timing and technique, drain use, postoperative complications, delirium, bed rest, and access to early rehabilitation [121]. Some factors are potentially modifiable, but observational associations with outcomes do not establish that changing them will improve recovery. Postoperative morbidity can prolong hospitalization and limit the patient’s ability to participate in rehabilitation. Prolonged postoperative bed rest may increase medical complications, whereas delirium is associated with longer hospitalization and may complicate mobilization and discharge planning [121,122].

Postoperative delirium can interrupt recovery even when the hematoma has been adequately evacuated. In the study by Ishida et al. [113], 27% of patients had worse functional status at discharge than before the onset of cSDH. Residual problems most often involved cognition or mobility. Older age, poorer pre-hospital function, and impaired consciousness were independently associated with poor recovery. Postoperative delirium was associated with longer hospitalization, but causality cannot be established from this retrospective design. Irrigation was associated with delirium in this retrospective cohort, but this finding does not establish that irrigation itself caused the complication.

A prospective multicenter study published in 2026 found postoperative delirium in 31.2% of 202 patients. Greater midline shift, higher pain intensity, electrolyte imbalance, and the use of restraint belts were independently associated with delirium. Age and hematoma thickness did not remain significant after adjustment [123]. Although pain, electrolytes, and restraint use may be modifiable, their estimated associations are not direct evidence that preventive interventions improve long-term cognition or mobility. Careful pain treatment, correction of electrolyte disorders, orientation, sleep protection, and avoidance of unnecessary physical restraints may reduce risk, although interventional studies are still needed.

Large observational cohorts provide further information on perioperative care. A prospective UK and Ireland study included 1205 patients treated across 26 neurosurgical units. Failure to insert a drain was independently associated with recurrence and unfavorable functional outcome. Older age, postoperative bed rest, and use of a single burr hole were associated with worse discharge functional outcome in this observational cohort. This outcome-specific association does not overturn randomized and meta-analytic comparisons showing no consistent recurrence advantage for a greater number of burr holes. High-flow oxygen and preoperative antiplatelet use were not independently associated with outcome [124]. Although observational data cannot prove causality, the results supported drain placement and questioned the routine use of enforced postoperative bed rest.

These findings need to be placed in the context of newer trials and meta-analyses. Drain use has a strong evidence base for reducing recurrence, while the superiority of one specific drain position or surgical configuration remains less certain [67,72]. In contrast, postoperative complications and immobility have direct effects on the patient’s ability to participate in rehabilitation. A technically minor difference in the operation may therefore matter less to functional recovery than delirium, infection, pain, or prolonged bed rest.

Timing may also affect recovery. Recent multicenter data suggest that unnecessary delays after the decision to operate may be associated with less favorable postoperative function [106]. However, treatment can be delayed for valid reasons, including reversal of anticoagulation, stabilization of comorbidities, or clarification of patient wishes. The relationship between delay and outcome may therefore be partly influenced by underlying clinical complexity.

Treatment-related predictors should not be considered separately from patient-related factors. A frail patient with baseline cognitive impairment is more susceptible to delirium, immobility, infection, and loss of independence. Conversely, early mobilization and coordinated geriatric or rehabilitation care may reduce the effect of this vulnerability. Recovery is therefore influenced not only by the procedure selected, but by the entire pathway from admission to discharge.

Taken together, randomized evidence most consistently supports postoperative drainage for recurrence prevention and suggests possible benefit from early mobilization; recommendations regarding delirium prevention and rehabilitation intensity are supported by less direct evidence. A subdural drain should be used when appropriate. Unnecessary bed rest should be avoided. Delirium risk should be assessed and modifiable factors addressed. Mobilization and discharge planning should begin early. These measures do not guarantee full recovery, but they may reduce preventable barriers to regaining independence.

The main patient-, hematoma-, and treatment-related factors that may influence recovery after cSDH are summarized in Table 5.

## 8. Clinical Assessment and Follow-Up

Clinical assessment should continue beyond the diagnosis and surgical procedure. It should establish whether the patient has returned to their previous cognitive and functional level. It should also identify complications, persistent disability, and the need for further support. This requires more than a neurological examination or a postoperative CT scan. Cognition, mobility, daily activities, frailty, and patient-reported recovery should also be considered [17,125].

There is currently no universally accepted cSDH-specific assessment battery or follow-up schedule. Earlier reviews found marked variation in the outcomes, instruments, and time points used across cSDH studies [125,126]. A practical approach should therefore be structured but flexible. It should reflect the patient’s symptoms, baseline function, treatment pathway, and personal goals.

### 8.1. Assessment Before Treatment

Pre-treatment assessment should define the patient’s usual level of function before the current deterioration. This information is necessary because many patients already have some degree of cognitive impairment, gait difficulty, frailty, or dependence. The assessment should determine what has changed and how quickly the change occurred [17].

A history from a relative, caregiver, or another person who knows the patient well is often essential. The patient may not recall a fall or may underestimate the extent of their decline. The collateral history should cover previous cognition, mobility, falls, self-care, medication management, living arrangements, and use of formal or informal support. It should also establish whether the patient was previously able to manage finances, travel independently, drive, or perform other complex activities [17,101,127]. The multidisciplinary and older-adult-specific cSDH guidance emphasizes the importance of documenting the patient’s previous physical and cognitive baseline [16,17].

Cognitive status should be assessed before treatment whenever the patient’s condition allows. A brief screening instrument such as the Montreal Cognitive Assessment (MoCA) or Mini-Mental State Examination (MMSE) may be used. However, neither test should be interpreted as a complete neuropsychological assessment. Acute pain, fatigue, medication effects, impaired consciousness, and delirium can all affect performance. A low score during the acute phase may therefore reflect several overlapping problems [13,43,95]. The MoCA is more sensitive than MMSE to some executive and mild cognitive deficits in other neurological populations, but neither has been specifically validated as a comprehensive cSDH recovery endpoint; original validation supports their general use [128,129].

Delirium should be considered separately from longer-standing cognitive impairment. Patients with fluctuating attention, altered alertness, disorganized thinking, or new confusion should be screened with a validated instrument such as the 4AT. The Confusion Assessment Method may be used where it forms part of local practice [16,17]. Delirium screening is particularly important because pre-existing dementia and acute cognitive dysfunction can occur together. The 4AT and Confusion Assessment Method have both undergone formal validation for rapid delirium detection in older inpatients [130,131].

Functional assessment should describe what the patient can actually do. The mRS offers a broad measure of global disability. An activities-of-daily-living scale, such as the Barthel Index, can provide more detail on feeding, dressing, toileting, transfers, and mobility. Premorbid function should be recorded separately from the patient’s status at presentation. Otherwise, a pre-existing disability may be incorrectly attributed to the hematoma [110,111,112,126]. The Barthel Index remains a widely used measure of basic activities of daily living [132].

Frailty should also be assessed using a validated instrument. The Clinical Frailty Scale is practical and is specifically mentioned in current cSDH guidance [16,17]. The score should reflect the patient’s usual state before the present illness rather than the temporary disability caused by the hematoma. Frailty assessment can help identify patients who may need geriatric review, nutritional support, delirium prevention, or more complex discharge planning [110,111]. The Clinical Frailty Scale was developed as a global clinical measure integrating comorbidity, function, and fitness [133].

Gait and balance should be evaluated when it is safe to do so. A basic examination should include transfers, standing balance, walking with or without assistance, and recent falls. Depending on the patient’s ability and local resources, this can be supplemented by gait speed, the Timed Up and Go test, or the Mini-Balance Evaluation Systems Test. Objective testing is useful because postural instability may not correlate closely with hematoma thickness or midline shift [12,98]. Formal walking tests should not be attempted in patients with reduced consciousness, severe weakness, or an immediate risk of falling. Timed Up and Go (TUG), the Mini-Balance Evaluation Systems Test (Mini-BESTest), habitual gait speed, and dual-task walking assess different constructs; general-population validity does not establish cSDH-specific responsiveness or minimal clinically important change [134,135].

In terms of feasibility, a simple timed 4 m gait-speed test and observed turns/transfers can be repeated with little equipment, while TUG adds standing, walking, turning, and sitting demands. Mini-BESTest provides a more detailed dynamic-balance assessment but requires staff training and adequate safety support. The most directly cSDH-specific objective evidence is the limited postural-sway work of Sundström et al. [12]; routine MoCA, MMSE, Barthel Index, TUG, and Mini-BESTest use in this context is largely supported by general neurological or geriatric validation and expert extrapolation rather than cSDH-specific measurement studies.

A practical pre-treatment assessment could therefore combine a brief cognitive screen, delirium screening, global disability, activities of daily living, frailty, and a basic mobility assessment. This proposed combination has not been validated as a mandatory cSDH battery. Its purpose is to create a multidimensional baseline that can be compared with the patient’s status after treatment [125,126,127].

### 8.2. Assessment After Treatment

Post-treatment assessment should begin during the early postoperative period and should use the same feasible measures recorded at baseline where possible. Neurological observations remain essential, but they should be accompanied by repeated evaluation of cognition, alertness, mobility, and daily function [136]. New confusion should prompt delirium screening. Lack of expected improvement should lead to a search for residual hematoma, recurrence, seizure, infection, metabolic disturbance, medication effects, or another neurological condition [16,62,137].

Current guidance recommends early mobilization and assessment of ongoing rehabilitation needs within 48 h after surgery [16]. This does not mean that every patient should follow the same mobilization schedule. Progress should reflect cardiovascular stability, neurological status, drain management, and fall risk. The GET-UP findings are discussed in Section 5.3; their applicability to individual patients depends on clinical stability and the specific rehabilitation setting [91,92].

Before discharge, the clinical team should document whether the patient has returned to their previous level of cognition and mobility. The assessment should include transfers, safe walking, need for supervision, ability to manage basic activities, and the availability of support at home. A favorable mRS score alone may not identify memory problems, poor balance, reduced endurance, or difficulty managing medication [19,90,98].

Cognition should be reassessed when the patient is medically stable and free from active delirium. A patient who performs poorly immediately after surgery may improve over the following days or weeks. However, persistent impairment is common. Persistent cognitive impairment has been described at three months and should prompt reassessment rather than the assumption of full recovery [13]. A practical approach is therefore to repeat cognitive and functional assessment during outpatient follow-up, particularly when deficits remain at discharge.

The optimal timing of outpatient reassessment has not been established. A pragmatic, explicitly proposed schedule is baseline/premorbid assessment, discharge, 6–12 weeks, and 6–12 months when deficits persist; these intervals are not a prospectively validated cSDH pathway [13,99]. Earlier review may be needed when the patient has ongoing symptoms, a high fall risk, difficult medication decisions, or limited support at home.

At baseline, record collateral premorbid cognition, walking aid, falls, frailty, and activities of daily living. At discharge, assess delirium, transfers, walking safety, and supports. At 6–12 weeks, reassess cognition, gait speed or TUG when safe, daily function, and patient-reported outcomes. At 6–12 months, persistent deficits justify re-evaluation for neurodegenerative, vascular, musculoskeletal, medication-related, and psychosocial contributors. This pathway is a pragmatic synthesis for multidisciplinary discussion, not a guideline-mandated monitoring schedule.

Follow-up should also address complex daily activities. Patients may appear neurologically improved but remain unable to drive, manage finances, prepare medication, or respond safely to unexpected situations. In one study, only 19 of 30 older patients who wished to resume driving met the required cognitive criteria. Some required repeated assessments over several months [101]. The study does not define universal driving rules, but it shows why symptom improvement alone may be insufficient before a patient returns to a demanding activity.

Patient-reported outcomes can add information that is missed by clinician-rated scales. Questions should cover memory, fatigue, mood, confidence when walking, perceived independence, and return to meaningful activities. In a 2025 study of 330 surgically treated patients, quality of life improved by 90 days among patients able to complete the assessment. However, inability to participate independently in the questionnaire was strongly associated with mortality [99]. This highlights an important limitation of patient-reported studies: the most vulnerable patients may be systematically underrepresented.

#### Follow-Up Imaging

Routine imaging should not replace clinical follow-up. In a randomized trial of 361 patients, scheduled CT at 2 and 30 days after surgery did not improve six-month survival without severe disability. It also did not improve other clinical outcomes. The routine-imaging group underwent more repeat procedures and generated higher medical costs [138].

These findings support a symptom-guided approach. Postoperative CT should be obtained when the patient deteriorates, develops a new neurological deficit, or fails to recover as expected [138]. The 2026 international consensus reached strong agreement on performing CT when postoperative recovery does not progress as expected. However, full consensus was not achieved on routine imaging in asymptomatic patients, which reflects continuing variation between healthcare systems [62].

A prospective study published in 2025 provided further support for clinically indicated imaging. Among 106 patients managed with a protocol-based approach, CT was mainly performed because of a new deficit, lack of early improvement, or later deterioration. The authors found no value in routine CT after an uncomplicated procedure in a clinically stable patient [139]. The study was conducted in a single low-resource setting and should not outweigh randomized evidence, but its findings are consistent with the broader literature.

Follow-up after conservative treatment is less standardized. A 2025 retrospective study found that few conservatively managed patients later crossed over to surgery. Deteriorating clinical status, rather than routine radiological surveillance, was the main reason for intervention [140]. The conservatively treated group was older and substantially frailer, so the results should not be applied to every patient. They nevertheless suggest that symptom-guided follow-up may be sufficient for many stable patients, provided that patients and caregivers receive clear safety-netting instructions [140].

Clinical follow-up should therefore remain central. Imaging is most useful when it answers a clinical question. A smaller hematoma on CT does not prove that memory, balance, or independence has returned.

### 8.3. When Further Care Is Needed

Further care is needed when the patient has not returned to their previous level of function or cannot be discharged safely. The type of support should reflect the main limiting problem. Some patients need further neurosurgical assessment. Others need geriatric medicine, rehabilitation, neuropsychology, or support within the community.

Geriatric assessment is particularly relevant for patients with frailty, multimorbidity, polypharmacy, recurrent falls, delirium, poor nutrition, or reduced independence. The ICENI guideline recommends specialist geriatric involvement for older or frail patients, with care guided by the principles of comprehensive geriatric assessment [16,17]. This approach examines medical conditions, cognition, function, medication, social circumstances, and treatment goals together.

The broader literature on traumatic brain injury in older adults supports a similar model. Management should account for premorbid health, frailty, cognitive reserve, pre-existing disease, rehabilitation potential, and the prevention of future falls. Decisions should be multidisciplinary and should include the patient and their family or advocates [127]. Although this literature is not specific to cSDH, its principles are highly relevant to the older cSDH population.

Rehabilitation referral should be considered when the patient has difficulty with transfers, standing balance, walking, endurance, or activities of daily living. Occupational therapy may be needed when cognition affects medication management, meal preparation, finances, or home safety. Persistent cognitive deficits may justify formal neuropsychological assessment. Speech and language therapy can be considered when communication or higher-level language problems remain.

Care transitions are an important source of risk. In a regional UK study, 41% of patients were repatriated to another hospital after surgery. Almost 40% of these patients developed a new complication after leaving the neuroscience unit, and many required institutional discharge or additional care. Communication difficulties and limited resources were recurring problems [141]. These findings support early discharge planning and a clear transfer of responsibility between neurosurgery, geriatric medicine, rehabilitation teams, primary care, and community services.

Every discharge summary should state the patient’s current cognitive and functional status. It should also include medication plans, antithrombotic management, rehabilitation needs, wound care, driving advice, follow-up arrangements, and instructions for obtaining urgent help [16,17]. Patients and caregivers should receive the same information in clear language.

Urgent reassessment is required when symptoms recur or worsen. Warning signs include new weakness, speech disturbance, increasing confusion, reduced consciousness, seizure, worsening headache, or a sudden decline in gait. These patients should undergo urgent neurological assessment and CT imaging [16]. Wound breakdown, fever, or an unexplained inflammatory response should also raise concern for postoperative infection.

Further care may also be needed when the patient appears medically stable but remains unable to resume important life roles. Problems with mood, confidence, social participation, caregiver burden, or return to work may not be visible during a brief neurosurgical appointment. Recent outcome reviews show that these domains are rarely measured despite their importance to patients [19,125]. Follow-up should therefore ask not only whether the hematoma has recurred, but also whether the patient has been able to return to a safe and meaningful daily life.

## 9. Current Evidence Gaps

Despite growing interest in cSDH, the evidence on cognitive and functional recovery remains limited. Only a small number of prospective studies have assessed cognition before and after treatment. The available studies used different screening tools, definitions of impairment, and follow-up periods. Premorbid cognitive status was often unknown, and appropriate control groups were uncommon [18]. As a result, the frequency, extent, and duration of cognitive recovery cannot yet be described with confidence.

A broader problem is the lack of consistent reporting across cSDH studies. Symptoms, baseline function, comorbidities, treatment details, and outcome measures have been recorded in different ways. Follow-up periods also vary considerably [125,126]. This heterogeneity makes direct comparisons difficult and limits the reliability of pooled analyses. Wider use of agreed core outcomes and common data elements would improve the quality and comparability of future research.

Most studies have focused on recurrence, mortality, radiological resolution, reoperation, and clinician-rated global disability. These measures remain important, but they provide only a partial view of recovery. Patient-reported cognition, mental health, quality of life, social participation, and return to previous activities are assessed much less often. Long-term follow-up is also uncommon, and the most frail or cognitively impaired patients may be excluded because they cannot complete standard questionnaires independently [19].

This limitation is also visible in recent MMAE research. Randomized trials and meta-analyses have mainly examined recurrence, treatment failure, reintervention, mortality, and the mRS [87,88]. Whether MMAE improves memory, gait, independence, or quality of life remains unclear. Future trials should therefore include structured cognitive and mobility assessments alongside patient- and caregiver-reported outcomes. Assessments should begin before treatment and continue beyond the early postoperative period. This would allow recovery to be described as a clinical and personal process, rather than only as the absence of hematoma recurrence.

As a narrative review, this article has certain inherent methodological limitations. The included studies differ in their designs, populations, outcome measures, and follow-up periods, which should be considered when interpreting and comparing their findings. Although the literature was selected with an emphasis on clinical relevance and methodological quality, some degree of selection bias cannot be excluded. Furthermore, the diversity of available evidence limits direct comparisons between interventions, particularly regarding cognitive and functional recovery. The proposed clinical assessment framework is therefore intended to support individualized patient care rather than represent a formally validated protocol. Long-term cohorts and patient-reported outcome studies may also be affected by survivor bias and nonresponse (responder-selection) bias, because patients who die, have more severe disability, or are unable to complete follow-up assessments may be underrepresented. Consequently, estimates of long-term recovery may not fully reflect the entire treated population. Future studies using standardized cognitive, mobility, and functional outcomes may help refine these recommendations and further improve the assessment of recovery following cSDH treatment.

## 10. Conclusions

cSDH is an increasingly common disorder in older adults and an important potentially reversible cause of cognitive and gait decline. Its presentation may be subtle. New confusion, cognitive slowing, gait instability, recurrent falls, or loss of independence should prompt consideration of cSDH, particularly when symptoms develop over days or weeks.

Treatment can lead to rapid neurological and cognitive improvement, but recovery is not always complete. Balance and mobility can improve after decompression; however, evidence for the optimal physiotherapy program or exercise dose remains insufficient, and some benefits attributed to rehabilitation may reflect spontaneous recovery. Radiological resolution should therefore not be assumed to represent full recovery.

Long-term studies show that some survivors continue to experience cognitive, functional, psychological, and social limitations years after treatment. Recovery should consequently be assessed across several domains, including cognition, gait, daily activities, independence, quality of life, and return to previous roles. These outcomes may follow different trajectories.

Optimal care extends beyond hematoma evacuation. It requires early recognition, assessment of premorbid function and frailty, appropriate treatment, prevention of complications, timely mobilization, and coordinated rehabilitation. Follow-up should focus on the patient’s actual cognitive and functional status rather than on imaging or a single global disability score alone. A multidisciplinary, patient-centered approach provides a rational framework for assessing meaningful recovery, although prospective studies must test whether the proposed assessment and rehabilitation pathway improves outcomes.

## Figures and Tables

**Table 1:** Practical clinical clues that should raise suspicion of cSDH.

Clinical Clue	Pattern Increasing Suspicion
New cognitive decline	Change over days or weeks; confusion, slowing, memory problems, or apathy.
New gait instability or falls	Unsteadiness, shuffling, reduced walking speed, or recurrent falls.
Focal or fluctuating symptoms	Weakness, speech disturbance, seizures, drowsiness, or transient deficits.
Relevant risk context	Recent fall or minor trauma, antithrombotic therapy, frailty, or alcohol misuse.
Loss of independence	New need for help with walking, self-care, medication, or daily activities.

**Table 2:** Selected evidence on cognitive/mobility recovery and MMAE, including non-equivalent trial endpoints.

Source and Design	Sample/Comparison/Assessment	Main Outcome, Follow-Up, Interpretation
Blaauw et al. [18]—systematic review	22 studies; 4 prospective pre/post cognition series; variable tests and timing	Presenting cognitive complaints/impairment 45% (95% CI 36–54%); tested impairment 61% pre- vs. 18% post-surgery (95% CIs 51–70% and 8–32%). Heterogeneous tests; unknown premorbid status.
Sundström et al. [12]—observational	22 cSDH patients vs. 58 controls; standing and walking sway; postoperative n = 5	Static and dynamic postural control impaired before surgery; apparent early improvement in small postoperative subset. No validated individual change threshold.
EMBOLISE [84]—randomized trial	n = 197 MMAE + surgery vs. 203 surgery; liquid embolic; 90-day repeat surgery	8/197 (4.1%) vs. 23/203 (11.3%); RR 0.36 (95% CI 0.11–0.80). Procedure-related serious adverse events: 2%, including 2 disabling strokes. No demonstrated cognition/gait advantage.
MAGIC-MT [85]—randomized trial	n = 360 MMAE vs. 362 usual care; 78% had burr-hole surgery; 90-day symptomatic recurrence/progression	24/360 (6.7%) vs. 36/362 (9.9%); risk difference −3.3 pp (95% CI −7.4 to 0.8), *p* = 0.10. Distinct endpoint, not repeat surgery alone.
STEM [86]—randomized trial	n = 149 vs. 161 randomized (primary analysis n = 120 vs. 129); surgical/nonsurgical pathways; 180-day composite	19/120 (16%) vs. 47/129 (36%); OR 0.36 (95% CI 0.20–0.66). Composite includes imaging and clinical events; cannot directly compare with EMBOLISE.
MEMBRANE [89]—randomized trial	376 randomized (188 per arm); n-BCA + standard care vs. standard care; evaluable 146 vs. 131 at 6 mo	17/146 (11.6%) vs. 29/131 (22.1%); common OR 0.53 (90% CI 0.31–0.91). Missing endpoint data, 90% CI, imaging/surgical composite; no cognitive/gait primary endpoint.
GET-UP [91,92]—randomized trial	104 per arm; mobilization within 12 h vs. 48 h bed rest; medical events to discharge, GOSE at 1 year	Complications 20/104 (19.2%) vs. 36/104 (34.6%), *p* = 0.012; GOSE ≥ 5 at 1 year 78/102 (76.5%) vs. 59/101 (58.4%), adjusted OR 2.006 (95% CI 1.076–3.739). Open-label, single-center; no standardized exercise dose.
Early mobility systematic review [93]	4 studies, 622 patients; heterogeneous early versus delayed ambulation	Pooled differences in recurrence and postoperative complications were not significant; protocols, comparators, and follow-up varied. No evidence of equivalence.

**Table 3:** Patient-centered domains of recovery after cSDH treatment.

Domain	What to Assess	Possible Measure
Neurological status	Consciousness, focal deficits, speech, seizures.	GCS and neurological examination.
Cognition and delirium	Attention, memory, executive function, fluctuating confusion.	MoCA or MMSE; 4AT or CAM.
Mobility and balance	Transfers, standing, walking assistance, falls.	Gait observation; TUG or Mini-BESTest when safe.
Daily function and frailty	ADLs, premorbid independence, support needs.	Barthel Index, mRS, Clinical Frailty Scale.
Quality of life and participation	Mood, confidence, driving, exercise, social roles.	Patient/caregiver report; quality-of-life measure.

Abbreviations: ADLs, activities of daily living; CAM, Confusion Assessment Method; CFS, Clinical Frailty Scale; GCS, Glasgow Coma Scale; MMSE, Mini-Mental State Examination; MoCA, Montreal Cognitive Assessment; mRS, modified Rankin Scale; TUG, Timed Up and Go.

**Table 4:** Distinct domains of recovery after cSDH treatment.

Recovery Domain	Practical Meaning
Radiological recovery	Reduction or resolution of the hematoma
Neurological recovery	Improvement in consciousness, weakness, speech, or other focal signs
Cognitive recovery	Improvement in attention, memory, processing speed, and executive function
Functional recovery	Regaining safe mobility and independence in daily activities
Participation	Returning to driving, exercise, work, hobbies, and social roles

**Table 5:** Main factors influencing recovery after cSDH treatment.

Factor Category	Key Factors	Potential Impact on Recovery	Clinical Implication
Patient-related	Frailty, premorbid dependence, dementia, poor mobility, malnutrition, multimorbidity	Lower likelihood of regaining previous independence and greater need for rehabilitation or support	Assess biological vulnerability and premorbid function rather than age alone
Hematoma-related	Hematoma volume, midline shift, bilaterality, internal architecture, residual cavity	May influence neurological severity, acute deterioration, persistence, and recurrence	Interpret imaging together with symptoms and neurological status
Treatment-related	Surgical delay, drain use, postoperative delirium, complications, bed rest, access to rehabilitation	May delay mobilization, prolong hospitalization, and reduce functional recovery	Use drainage when appropriate, prevent delirium, avoid unnecessary bed rest, and begin mobilization early

## Data Availability

No new data were created or analyzed in this study. Data sharing is not applicable to this article.

## Abbreviations

The following abbreviations are used in this manuscript:

ADLs	activities of daily living
CAM	Confusion Assessment Method
CFS	Clinical Frailty Scale
cSDH	chronic subdural hematoma
CT	computed tomography
FIT-score	Functional Impairment after Burr-Hole Trepanation score
GCS	Glasgow Coma Scale
Mini-BESTest	Mini-Balance Evaluation Systems Test
MMAE	middle meningeal artery embolization
MMSE	Mini-Mental State Examination
MoCA	Montreal Cognitive Assessment
MRI	magnetic resonance imaging
mRS	modified Rankin Scale
TUG	Timed Up and Go
